# Comparison of Trihalomethanes in Tap Water and Blood: A Case Study in the United States

**DOI:** 10.1289/ehp.1104347

**Published:** 2012-01-26

**Authors:** Zorimar Rivera-Núñez, J. Michael Wright, Benjamin C. Blount, Lalith K. Silva, Elizabeth Jones, Ronna L. Chan, Rex A. Pegram, Philip C. Singer, David A. Savitz

**Affiliations:** 1 National Research Council, Washington, DC, USA; 2 National Center for Environmental Assessment, U.S. Environmental Protection Agency, Cincinnati, Ohio, USA; 3 Centers for Disease Control and Prevention, Atlanta, Georgia, USA; 4 Association of Schools of Public Health, Washington, DC, USA; 5 Department of Epidemiology, Gillings School of Global Public Health, University of North Carolina–Chapel Hill, Chapel Hill, North Carolina, USA; 6 National Health and Environmental Effects Research Laboratory, U.S. Environmental Protection Agency, Research Triangle Park, North Carolina, USA; 7 Department of Environmental Sciences and Engineering, Gillings School of Global Public Health, University of North Carolina–Chapel Hill, Chapel Hill, North Carolina, USA; 8 Departments of Community Health and Obstetrics and Gynecology, Brown University, Providence, Rhode Island, USA

**Keywords:** blood THM, blood–water correlations, brominated THMs, noningestion water activities, trihalomethanes

## Abstract

Background: Epidemiological studies have used various measures to characterize trihalomethane (THM) exposures, but the relationship of these indicators to exposure biomarkers remains unclear.

Objectives: We examined temporal and spatial variability in baseline blood THM concentrations and assessed the relationship between these concentrations and several exposure indicators (tap water concentration, water-use activities, multiroute exposure metrics).

Methods: We measured water-use activity and THM concentrations in blood and residential tap water from 150 postpartum women from three U.S. locations.

Results: Blood ΣTHM [sum of chloroform (TCM), bromodichloromethane (BDCM), dibromo-chloromethane (DBCM), and bromoform (TBM)] concentrations varied by site and season. As expected based on variable tap water concentrations and toxicokinetic properties, the proportion of brominated species (BDCM, DBCM, and TBM) in blood varied by site (site 1, 24%; site 2, 29%; site 3, 57%) but varied less markedly than in tap water (site 1, 35%; site 2, 75%; site 3, 68%). The blood–water ΣTHM Spearman rank correlation coefficient was 0.36, with correlations higher for individual brominated species (BDCM, 0.62; DBCM, 0.53; TBM, 0.54) than for TCM (0.37). Noningestion water activities contributed more to the total exposure metric than did ingestion, but tap water THM concentrations were more predictive of blood THM levels than were metrics that incorporated water use.

Conclusions: Spatial and temporal variability in THM concentrations was greater in water than in blood. We found consistent blood–water correlations across season and site for BDCM and DBCM, and multivariate regression results suggest that water THM concentrations may be an adequate surro-gate for baseline blood levels.

Although water disinfection is essential for reducing the risk of pathogens in the public water supply, potentially harmful disinfection by-products (DBPs) such as trihalomethanes (THMs) can be formed by the reaction of chlorine and other disinfectants with naturally occurring organic matter and inorganic chemi-cals in the water. The presence of THMs in the domestic water supply results in widespread exposure from activities such as water consumption, bathing, showering, and swimming (Ashley et al. 2005; Backer et al. 2000; Cammann and Hubner 1995). Exposure to DBPs has been associated with an increased risk of bladder cancer (Cantor et al. 1987, 2010; McGeehin et al. 1993; Villanueva et al. 2007). Although the evidence is not as strong, a recent meta-analysis on 13 different studies suggested a small increased risk of colon (relative risk (RR), 1.27; 95% confidence interval (CI): 1.08, 1.50) and rectal (RR, 1.30; 95% CI: 1.06, 1.59) cancer was associated with DBP exposures through drinking water (Rahman et al. 2010). DBP exposure has also been associated with fetal growth but not with preterm delivery, as noted in a recent meta-analysis by Grellier et al. (2010). Most of the previous studies have shown a small but consistent increased risk of small for gestational age/intrauterine growth retardation with increasing THM exposures (Bove et al. 1995; Dodds et al. 1999; Hoffman et al. 2008; Porter et al. 2005; Wright et al. 2004; Yang et al. 2007), and three of four studies suggested an increased risk of stillbirth (Dodds et al. 1999, 2004; King et al. 2000; Toledano et al. 2005). There is also suggestive evidence of an association between DBP exposure with some cardiac defects (Hwang et al. 2008), but the evidence is not as strong for other outcomes such as miscarriages (Savitz et al. 2006; Waller et al. 2001). One of the limitations of previous epidemiological studies is uncertainty in the exposure measures used to characterize DBP mixtures of interest during the relevant critical periods of exposure.

THMs are usually the most abundant class of DBPs found in chlorinated drinking water in the United States (Krasner et al. 2006). THM concentrations correlate with levels of some other types of halogenated DBPs, allowing THMs to be used as surrogate markers of DBPs (Obolensky and Singer 2005). However, the distribution among the four different THM species [chloroform (TCM), bromodichloro-methane (BDCM), dibromo-chloromethane (DBCM), and bromoform (TBM)] can vary appreciably, depending on the concentration of bromide in the water at the time chlorine is added for disinfection (Obolensky and Singer 2005). Exposure assessment for THMs is complicated by this variation in the speciation of the four THMs, multiple routes of exposure (i.e., ingestion, inhalation, dermal absorption), intra- and interindividual variability in behavior, and interindividual physio-logical differences in absorption, distribution, metabolism, and excretion of the four THMs (Backer et al. 2000, 2008; Leavens et al. 2007). Blood and expired air biomarkers of THM exposure can be used to estimate internal dose (Gordon et al. 2006; LaKind et al. 2010; Weisel and Jo 1996), but these measures are strongly influenced by very recent exposures. For example, Lynberg et al. (2001) reported significant increases in blood THM levels after showering, while Nuckols et al. (2005) found that the greatest influence on blood THM concentrations was due to showering, bathing, and washing dishes by hand. Morning blood samples collected before any major water-use activity can be expected to yield baseline THM concentrations, as experimental studies have shown that pre-exposure breath levels of TCM were reached 4 hr after inhalation and dermal exposures during showering or bathing (Weisel and Jo 1996) and that baseline blood BDCM levels were attained approximately 4 hr after oral administration and 6–7 hr after dermal exposure (Leavens et al. 2007). Several factors may influence baseline blood THM measurements: *a*) DBP concentrations based on formation dynamics in water distribution systems; *b*) exposures from sources other than water; *c*) ambient indoor air concentrations of each THM species (volatility decreases in the order: TCM > BDCM > DBCM > TBM); *d*) body burden and the relative timing of exposure; *e*) partitioning of each individual THM species between body fat and the blood, which is in turn affected by the lipophilicity of each THM (lipophilicity decreases in the order: TBM > DBCM > BDCM > TCM); *f* ) personal charac-teristics and behavior (e.g., smoking, alcohol use, water-use patterns); and *g*) genetic and physiological differences that can affect THM metabolism (Blount et al. 2011).

Although some studies have identified important activities that contribute to peak blood THM levels, few studies have examined predictors of baseline exposures. Additionally, the relationship between blood THM levels and less direct measures of THM exposures has not been well characterized in an epidemiological study. To further inform exposure assessment efforts, we examined specific water-use activities measured from 24-hr diary data in relation to THMs in blood from 150 postpartum women. The primary objectives of this study were to describe temporal and spatial variability in blood THM biomarkers and to assess the relationship between these blood THM biomarkers, water concentrations, specific water-use activities, multiroute exposure metrics, and other covariates.

## Materials and Methods

*Selection of sites and participant recruitment.* Right from the Start (RFTS) was a prospective cohort study of drinking water DBPs and pregnancy health conducted in three metro-politan areas of the United States (Savitz et al. 2005, 2006). We selected these locations to include a wide range of individual DBP species and summary measures such as the sum of TCM, BDCM, DBCM, and TBM (ΣTHM). Sites 1 and 3 had moderate levels of chlorinated and brominated DBPs, respectively, and we chose them because they used chloramination rather than free chlorine for terminal disinfection. Chloramination results in minimal additional DBP formation within the distribution system; therefore, we would have expected all of the study participants within the same site to have similar tap water DBP concentrations for samples collected within the same week (Singer 1994). Site 2 used free chlorine for the distribution system, but THM levels were so low that all consumers were exposed to low THM levels from residential tap water.

To be eligible to participate in the RFTS study, women had to be ≥ 18 years of age, reside and remain in one of the three metropolitan areas, use public drinking water, be able to speak and write English or Spanish, not have used assisted reproductive technology, and be trying to become pregnant or pregnant at < 12 weeks of gestation, with the intent to carry the pregnancy to term (Promislow et al. 2004). Postpartum women who had participated in the RFTS study and were at least 30 days past delivery, not pregnant at the time of screening and enrollment, still residing in the study areas, and using public drinking water were eligible for the study of blood THMs. The institutional review boards at the University of North Carolina–Chapel Hill, University of Tennessee, and University of Texas approved the study protocols, and participants gave informed consent. Among the total of 238 women that were eligible and agreed to participate, 153 (64%) provided blood and water samples [see Supplemental Material, Figure 1 (http://dx.doi.org/10.1289/ehp.1104347)]. The participation rate was 76% for site 1 and 57% for sites 1 and 2. To better assess seasonal variation in DBP levels, we collected water and blood samples from a subset of women in the summer and winter from site 1 (*n* = 29) and site 3 (*n* = 2). Blood and water samples were collected from January to March of 2004 (winter) for site 1, June to August of 2004 (summer) for sites 1–3, and December 2004 to January 2005 (winter) for site 3.

*Blood and water sample collection and analysis.* Trained personnel scheduled morning home visits to collect blood and tap water samples before the participants had any contact with water. After signing a consent form, trained technicians (phlebotomists) collected a 10-mL blood sample from each participant via venipuncture into gray-top glass tubes (Vacutainer® Becton Dickinson, Franklin Lakes, NJ) that were specially treated before use to remove background THM contamination (Cardinali et al. 1995). We mixed the blood samples to dissolve the anticoagulant immediately after the blood draw. The technicians collected a 12-mL water sample during the same home visit from a nonaerated, cold water tap. We kept all blood and water samples in coolers until they were shipped to the Centers for Disease Control and Prevention for analysis. We collected a total of 184 blood and water samples from 153 study participants. Seventy-four women provided blood samples in summer only, 48 in winter only, and 31 in both summer and winter. We excluded blood and water samples (*n* = 4) from two participants from the analysis because of laboratory data quality concerns, and we excluded the water and blood samples for another participant who was exposed through a key water-use activity within 1 hr of sampling. In addition, we did not examine four water samples because of unacceptable headspace volume and/or freezing of vials. A total of 179 blood samples and 175 water samples from 150 women were available for analysis [see Supplemental Material, Figure 1 (http://dx.doi.org/10.1289/ehp.1104347)].

Isotope-dilution–based quantification of THM concentrations in tap water and blood samples was accomplished using solid--phase microextraction/gas chromatography (SPME/GC) with mass spectrometry (MS) (Cardinali et al. 2004) and high-resolution MS (Bonin et al. 2005), respectively. We added stable isotopically labeled analogs of the compounds of interest to 3 g blood and 5 mL water and sealed each sample in a 10-mL headspace vial. We heated (30°C for blood and 50°C for water) and agitated (350 rpm for blood and 500 rpm for water samples) samples using a CTC CombiPal® SPME autosampler (LEAP Technology, Carrboro, NC) to facilitate extraction of volatiles from the sample headspace onto an SPME fiber (Carboxen/PDMS, Supelco, Bellefonte, PA). After extraction, we inserted the fiber into a hot GC (5890 Series II; Agilent Technologies, Santa Clara, CA) inlet to desorb volatile compounds that were resolved chromatographically and then quantified in a high-resolution MS (Thermo Finnigan MAT 95; Thermo Finnigan, San Jose, CA) for blood and a quadrupole MS for water (Trace MS; Thermo Finnigan). Final quantification was based on daily seven-point calibration curves, and we normalized the concentrations according to sample weight. The limit of detection (LOD) in water was 0.93 µg/L for TCM, 0.21 µg/L for BDCM, 0.49 µg/L for DBCM, and 0.15 µg/L for TBM. The LOD in blood for TCM was 2.2 ng/L, 0.24 ng/L for BDCM, 0.21 ng/L for DBCM, and 0.58 ng/L for TBM. Out of 175 water samples, 5% were below the LOD for TCM, 2% for BDCM, 3% for DBCM, and 18% for TBM. Out of 179 blood samples, 6% were below the LOD for TCM, 8% for BDCM, 18% for DBCM, and 59% for TBM. Concentrations below the LOD were replaced with LOD/_√_-2 (Hornung and Reed 1990) for the analyses.

*Data collection.* The participants self--administered a water-use activity diary 24 hr before the home visit, and we reminded them not to have any contact with water for at least 4 hr before their home-visit appointment. The 24-hr diary included information on water consumption practices (e.g., use of filters and other point-of-use devices), time, duration and location of showering and/or bathing, time spent bathing children, time spent washing dishes (and glove use), use of swimming pools, and use of fans and opening of windows while showering/bathing one’s self or children. Sociodemographic data collected from the main epidemiological study (Savitz et al. 2005) were also available for this population [see Supplemental Material, Table 1 (http://dx.doi.org/10.1289/ehp.1104347)].

As part of the diary, we asked study partici-pants how many bottles of water and glasses/cups of cold tap water, hot tap water, and tap-water–based beverages (including juice, coffee, tea, and other beverages made from tap water) they consumed each day. We also asked that participants define their glass or cup sizes according to three options: small (0.1–0.3 L), medium (> 0.3–0.6 L), or large (> 0.6–1.0 L) for cold tap water beverages and small (0.1–0.3 L), medium (> 0.3–0.5 L), or large (> 0.5–0.7 L) for hot tap water beverages. We used the midpoint for each size range to estimate water consumption in ounces per day. We converted bottled water intake (spring water, mineral water, distilled water, sparkling water, or any water purchased in bottles or plastic jugs or obtained from a water cooler) to liters based on reported container sizes: small (8–12 ounces), medium (14–24 ounces), and large (26–34 ounces).

*Exposure assessment.* To help assess the primary determinants of blood THM levels, we developed a total daily exposure metric based on the main activities that impact ingestion, inhalation, and dermal absorption. We used six activities to calculate the daily exposure metrics for the 24 hr before sampling: *a*) total tap water intake (liters), *b*) total time showering/bathing themselves (minutes), *c*) total time showering/bathing children (minutes), *d*) total post-shower/bathroom time (minutes), *e*) total time washing dishes (minutes), and *f* ) total time swimming (minutes). We summed intake of tap water and tap-water–based beverages to estimate ingestion exposure to THMs, and we used the reported other activities to estimate noningestion exposures. We applied a reduction of 70% in THM levels to the ingestion estimate for hot beverages, a 50% reduction for point-of-use filtration to filtered tap water, and a 50% reduction to those who reported using gloves while washing dishes (Forssén et al. 2007; Krasner and Wright 2005). We did not include bottled water consumption in the analy-sis because it typically contains very low levels of THMs (Weinberg et al. 2006). Although we did not integrate data on the use of fans and the opening of windows/doors during showering/bathing because of the uncertainty associated with these specific practices on DBP levels (e.g., post-shower/bath levels), we performed sensitivity analyses (assuming 75% reduction) to assess the potential impact of these exposure modifying factors on the total estimate of ΣTHM exposure.

To integrate equivalent THM dose contributions from different exposure routes, we calculated a total exposure metric based on the summation of ingestion and noningestion activities. To allow for a common metric across disparate activities, we used liter--equivalents based on human biomonitoring data collected during controlled dermal or inhalation studies of TCM (Kerger et al. 2000; Weisel and Jo 1996) and previously applied in epidemiological studies (Dodds et al. 2004; King et al. 2004). The equivalency scores, including reductions applied to the afore-mentioned exposure modifying factors, were based on a presumed dose equivalency of 1 L total tap water intake, 5-min shower/bath, 15-min shower/bath for children, 15-min post-shower/bath time spent in the bathroom, 15 min of washing dishes by hand, and a 5-min swim. We examined ingestion and noningestion THM equivalency score tertiles in relation to blood THM concentrations. We restricted this analysis (*n* = 150) to the first reported water diary entry for women with both summer and winter measurements (68% summer, 32% winter). Given potential toxicokinetic differences between specific compounds, we performed sensitivity analyses to assess the effect of estimated equivalencies (e.g., a 2-min shower/bath = 1 L ingested water) on the total exposure metric results.

*Statistical analysis.* We conducted statistical analyses using SAS statistical software (version 9.2; SAS Institute Inc., Cary, NC). We calculated descriptive statistics for blood, tap water, and sociodemographic characteristics of the study participants. We defined ΣTHM as the sum of TCM, BDCM, and DBCM, and TBM, concentrations in water (micrograms per liter) and blood (nanograms per liter). We defined brominated high-resolution THMs as the sum of BDCM, DBCM, and TBM in these two media. We did not weight the sums according to bromide content. Descriptive tests (skewness, kurtosis), histograms, and normal probability plots revealed deviations from a normal distribution for DBPs in blood and water [see Supplemental Material, Figures 2 and 3 (http://dx.doi.org/10.1289/ehp.1104347)]; therefore, data were log_10_ transformed for the regression models and analysis of variance (ANOVA). We calculated the percentages of the brominated species in tap water and blood samples using the geometric means (GMs) for individual THM species and the ΣTHM GM for each site and season. We used Spearman rank correlation coefficients (*r*_S_) to quantify the correlation between tap water and blood ΣTHM concentrations, between sites and seasons, and across individual THMs. We used paired *t*-tests to compare mean water and blood THM concentrations between different seasons of sample collection. Because of the small sample size and limited number of samples, we restricted the intraindividual variability analysis to the 29 site 1 participants with repeated measures. We used linear regression to estimate the change in ΣTHM in blood per unit increase of ΣTHM in water. We adjusted the regression models for maternal age, ethnicity, education, smoking, marital status, body mass index, household income, season, study site, and reported water-use activities. We selected confounders based on percent change (> 10%) in regression coefficients from the univariate models. We performed trend analyses using one-way ANOVA to evaluate blood THM concentrations across the ingestion, noningestion, and total exposure metric tertiles. We defined statistical significance as a *p*-value < 0.05 for the regression models, Spearman rank correlations, and ANOVAs.

## Results

The study participants were predominantly married (78%), Caucasian (69%), and between 25 and 34 years of age [67%; see Supplemental Material, Table 1 (http://dx.doi.org/10.1289/ehp.1104347)]. Study participants commonly consumed tap water (71%), and 21% of tap water users reported exclusive use of filtered tap water for consumption (data not shown). For site 1, the GM tap water ΣTHM level was notably higher in summer (46.3 µg/L; *n* = 47) than in winter (25.2 µg/L; *n* = 50; *p* < 0.01; Table 1). Blood ΣTHM concentrations were also higher in summer (GM = 26.2 ng/L; *n* = 47) than in winter (GM = 15.9 ng/L; *n* = 49) for site 1 (*p* < 0.01). Although site 2 had low tap water ΣTHM concentrations (GM = 4.8 µg/L; *n* = 49), the GM for blood ΣTHM (12.6 ng/L; *n* = 49) was approximately 60% of the GMs for sites 1 and 3. Higher tap water ΣTHM levels in winter (GM = 28.5 µg/L) were detected in site 3 than in the limited number of summer samples (GM = 14.6 µg/L). The brominated species in tap water were predominant in site 2 (75%; summer samples only) and site 3 (68%; summer and winter samples) but not in site 1 (35%; summer and winter samples). The proportion of brominated species detected in blood was 24% for site 1, 29% for site 2, and 57% for site 3.

**Table 1:** Tap water and blood THM concentrations by season and site for 150 postpartum women.^*a*^

Water concentration (μg/L)b							Blood concentration (ng/L)c						
THM	n	GM (GSD)	Median	Range	n	GM (GSD)	Median	Range
Site 1																
Overall																
TCM		97		21.3 (2.4)		31.0		0.18–65.0		96		14.6 (1.8)		14.5		1.5–81.0
BDCM		97		8.5 (2.5)		12.0		BLD–17.0		96		3.0 (1.8)		3.3		0.44–17.0
DBCM		97		3.1 (2.1)		3.0		BLD–7.1		97		1.2 (1.7)		1.2		0.44–8.6
TBM		97		0.19 (1.5)		0.14		BLD–0.80		97		0.77 (1.3)		0.72		0.71–2.4
ΣTHM		97		33.8 (2.2)		47.3		0.38–86.1		96		20.3 (1.7)		21.1		3.1–89.8
Winterd																
TCM		50		16.4 (3.9)		23.0		0.18–65.0		49		11.7 (2.3)		15.7		1.5–81.0
BDCM		50		6.0 (3.6)		8.9		BLD–17.0		49		2.1 (2.2)		2.7		0.44–13.0
DBCM		50		1.9 (2.6)		2.4		BLD–4.0		50		0.74 (1.8)		0.75		0.44–4.3
TBM		50		0.09 (1.3)		0.10		BLD–0.15		50		0.73 (1.1)		0.10		0.71–1.2
ΣTHM		50		25.2 (3.4)		34.6		0.38–86.1		49		15.9 (2.1)		18.0		3.1–89.8
Summer																
TCM		47		28.1 (2.4)		34.0		0.1–48.0		47		18.3 (1.8)		18.0		5.3–61.0
BDCM		47		12.2 (2.5)		15.0		0.18–17.0		47		4.6 (1.8)		4.3		0.85–17.0
DBCM		47		5.3 (2.1)		6.4		0.17–7.1		47		2.0 (1.7)		1.9		0.44–8.6
TBM		47		0.42 (1.5)		0.47		BLD–0.80		47		0.82 (1.3)		0.71		0.71–2.4
ΣTHM		47		46.3 (2.2)		55.5		0.77–72.4		47		26.2 (1.7)		24.8		8.1–82.9
Site 2																
Summer																
TCM		49		0.83 (1.0)		0.90		BLD–4.4		49		6.9 (3.4)		5.4		1.5–130.0
BDCM		49		1.3 (2.0)		1.4		0.19–5.2		49		1.1 (2.1)		1.1		0.44–7.4
DBCM		49		1.6 (1.9)		1.6		0.32–7.0		49		1.1 (2.1)		1.2		0.44–10.0
TBM		49		0.69 (0.8)		0.66		0.15–8.0		49		1.4 (2.0)		1.3		0.71–18.0
ΣTHM		49		4.8 (1.9)		4.6		0.83–19.5		49		12.6 (2.6)		10.7		3.1–132.8
Site 3																
Overall																
TCM		29		5.2 (10.5)		9.6		BLD–85.0		33		8.6 (2.2)		8.6		1.5–47.0
BDCM		29		6.3 (12.0)		18.0		BLD–66.0		33		5.5 (2.1)		5.5		1.3–30.0
DBCM		29		8.1 (7.3)		19.0		BLD–41.0		33		5.6 (2.0)		6.3		1.5–27.0
TBM		29		2.5 (3.9)		4.1		BLD–9.5		33		2.1 (1.8)		1.8		0.71–7.8
ΣTHM		29		24.8 (7.6)		52.7		BLD–197.2		33		23.1 (1.9)		22.8		6.1–107.8
Winter																
TCM		23		5.8 (10.3)		12.0		BLD–85.0		27		8.7 (2.4)		8.6		1.5–47.0
BDCM		23		7.3 (10.7)		18.0		BLD–66.0		27		5.6 (2.2)		5.5		1.3–30.0
DBCM		23		9.3 (6.0)		18.0		BLD–41.0		27		5.7 (2.1)		6.4		1.5–27.0
TBM		23		2.7 (3.2)		4.1		BLD–7.2		27		2.0 (1.8)		1.8		BLD–7.8
ΣTHM		23		28.5 (6.5)		52.5		BLD–197.2		27		23.5 (2.0)		22.8		6.1–107.8
Summer																
TCM		6		3.2 (12.9)		8.7		BLD–45.0		6		8.1 (1.6)		8.7		4.6–14.0
BDCM		6		3.4 (21.1)		19.5		BLD–32.0		6		4.7 (1.9)		5.6		2.0–9.6
DBCM		6		4.8 (16.1)		26.0		BLD–34.0		6		5.2 (1.9)		6.3		1.6–9.1
TBM		6		1.9 (7.9)		6.0		BLD–9.5		6		2.4 (1.8)		2.6		1.2–4.8
ΣTHM		6		14.6 (14.3)		65.0		BLD–104.8		6		21.8 (1.5)		25.7		12.2–30.6
Abbreviations: BLD, below the LOD (replaced with LOD/√_2 for the analysis); GSD, geometric standard deviation. aSeventy-four women provided samples in summer, 48 in winter, and 29 in summer and winter. bNine water samples were not examined because of unacceptable headspace volume and/or freezing of vials. cFour blood samples were excluded because of laboratory data quality concerns. dOne blood sample failed laboratory quality controls for TCM, BDCM, and ΣTHM for site 1 in winter.																

The overall Spearman rank correlation coefficient for ΣTHM concentrations in tap water and blood was 0.36 (*p* < 0.01) based on the first measurement for each study participant [*n* = 150; see Supplemental Material, Table 2 (http://dx.doi.org/10.1289/ehp.1104347)]. Despite differences in the relative proportion of brominated THMs across the different matrices, we found stronger and more consistent correlations for BDCM, DBCM, TBM, and the sum of the brominated THMs (*r*_S_ = 0.53–0.62) than for TCM (*r*_S_ = 0.37). The correlation between water and blood ΣTHM concentrations was considerably higher in site 3 (*r*_S_ = 0.51) than in site 1 (*r*_S_ = 0.12) or site 2 (*r*_S_ = –0.04). The 29 participants who had both winter and summer samples from site 1 had higher blood ΣTHM concentrations in summer (GM = 26.1 ng/L) than in winter (GM = 12.3 ng/L; *p* < 0.01; data not shown). The correlation coefficient between blood and water ΣTHM was 0.43 in winter and –0.06 in summer among the 29 participants with repeated measures. The correlation coefficients between the two blood and two tap water samples were 0.57 and –0.12, respectively.

Blood ΣTHM concentration was lower (GM = 15.6 ng/L) in the first noningestion tertile than in the second (20.0 ng/L) and third tertiles (21.2 ng/L; Table 2; *p*-value for trend = 0.15). Blood ΣTHM concentrations also tended to increase across the ingestion tertiles (data not shown). The gradient in blood ΣTHM concentrations for the total exposure metric derived from liter-equivalent estimates was comparable with that for noningestion water activities, suggesting little influence from ingestion (Table 2). Sensitivity analyses showed minimal changes in blood THM concentrations (2–3%) based on 75% reduction in DBP levels during the post-shower/bathing time due to use of fans or opening of windows/doors. Additional sensitivity analyses using the showering/bathing equivalency of 10 or 15 min per 1 L of ingested water (while keeping other parameters constant) showed minimal impacts (4–11%) on mean blood THM levels and similar trends (as the main results) across the tertiles. Given that Leavens et al. (2007) indicate a greater role for dermal absorption for the brominated compounds than for TCM, we conducted sensitivity analyses to examine the contribution of activities with heavy dermal exposures. Therefore, we separately examined the impact of changing the swimming and showering/bathing equivalent to both 1 min and 2 min per 1 L ingestion, respectively. Although the lowest blood mean levels were consistently found among the lowest exposure tertiles, monotonic increases were not as evident across the tertiles as those found for the main study results. Univariate linear regression analysis of the first samples collected per subject showed that tap water ΣTHM concentration was the strongest predictor of blood ΣTHM levels among the water-use indicators; for a 1-µg/L increase in ΣTHM water levels, blood ΣTHM levels increased by 0.21 ng/L (*p* < 0.01). The change in blood levels was 0.19 ng/L per 1-µg/L increase in ΣTHM tap water concentrations (*p* < 0.05) after adjustment for confounding, with ΣTHM water concentration, education, and marital status identified as the strongest predictors of blood ΣTHM concentrations [see Supplemental Material, Table 3 (http://dx.doi.org/10.1289/ehp.1104347)].

**Table 2:** The influence of noningestion exposure and total exposure metrics on blood ΣTHM concentrations for 150 postpartum women.

Noningestion exposures (ng/L)a					Total exposure (ng/L)b				
Exposure metric	GM (GSD)	Median	Range	GM (GSD)	Median	Range
TCM												
Tertile 1		9.0 (4.3)		8.6		1.5–61.0		10.1 (2.3)		9.8		1.5–61.0
Tertile 2		10.7 (3.1)		12.0		1.5–130.0		9.6 (2.1)		12.0		1.5–130.0
Tertile 3		12.6 (5.3)		13.0		1.5–81.0		12.4 (1.4)		13.0		1.5–81.0
BDCM												
Tertile 1		2.0 (1.2)		2.2		BLD–15.0		2.3 (2.1)		2.5		BLD–15.8
Tertile 2		3.3 (1.5)		3.6		BLD–15.8		3.0 (2.3)		3.8		BLD–17.0
Tertile 3		2.7 (1.4)		2.8		BLD–30.0		2.6 (1.4)		2.7		BLD–30.0
DBCM												
Tertile 1		1.4 (1.7)		1.3		BLD–9.7		1.5 (0.9)		1.4		BLD–14.0
Tertile 2		2.1 (2.2)		2.1		BLD–14.0		2.0 (0.7)		2.1		BLD–9.7
Tertile 3		1.9 (1.4)		1.7		BLD–27.4		1.8 (1.3)		1.7		BLD–27.0
TBM												
Tertile 1		1.1 (0.9)		0.7		BLD–16.0		1.1 (1.2)		0.7		BLD–16.0
Tertile 2		1.2 (0.8)		1.1		BLD–18.0		1.2 (1.9)		1.0		BLD–18.0
Tertile 3		1.2 (1.4)		0.9		BLD–7.8		1.2 (0.8)		1.1		BLD–7.8
Brominated THMs												
Tertile 1		5.2 (2.1)		4.7		1.6–22.3		5.5 (2.4)		5.0		1.6–30.4
Tertile 2		7.3 (1.2)		7.4		1.6–30.4		6.9 (3.9)		7.4		1.9–27.2
Tertile 3		6.3 (1.3)		5.7		1.6–60.8		6.1 (3.2)		6.0		1.6–60.8
ΣTHM												
Tertile 1		15.6 (8.6)		17.7		3.1–82.9		17.0 (3.8)		17.7		3.6–82.9
Tertile 2		20.0 (7.5)		21.5		4.0–132.8		18.6 (4.1)		22.5		3.1–132.8
Tertile 3		21.2 (10.8)		22.2		3.1–107.8		20.7 (3.4)		21.2		3.1–107.8
aNoningestion exposures: showering/bathing, bathing children, postshower/bathroom time, washing dishes by hand, and swimming. Exposure categories are based on the tertiles of the sum (minutes) of the noningestion exposures with the following cut points: tertile 1, ≤ 33 min; tertile 2, > 33–59 min; tertile 3, > 59 min. bTotal exposure includes ingestion and noningestion exposures. Equivalencies: ingestion exposure = 1 L total tap water, 5 min. Noningestion exposures: shower/bathing, 5 min; bathing children, 15 min; postshower/bathroom time, 15 min; washing dishes, 15 min; swimming, 5 min (70% reduction was applied to hot beverages and 50% when point-in-use filtration or wearing gloves while washing the dishes was reported). Exposure categories were based on the tertiles of equivalency scores with the following cut points: tertile 1, score ≤ 4.58; tertile 2, score 4.59–6.87; tertile 3, score > 6.87.												

## Discussion

Consistent with previously reported studies showing THM seasonality in water (Nieminski et al. 1993; Parvez et al. 2011; Williams et al. 1997), we found higher THM concentrations in summer tap water samples than in winter samples among site 1 participants. Similarly, mean baseline blood THM levels among site 1 participants were higher in summer than in winter. Overall, we saw moderate correlations between blood and tap water concentrations of ΣTHM and all individual THMs; however, this was largely due to stronger correlations in site 3 (*r*_S_ = 0.51). Despite differences in the relative proportion of brominated compounds across the different matrices, water concentration appeared to be a relatively good marker (*r*_S_ = 0.53) of baseline brominated blood THM levels. We also noted fairly consistent blood–water correlations for BDCM and DBCM (*r*_S_ = 0.26–0.57) across the three sites despite some seasonal differences. The TCM blood–water correlations, however, were more varied (–0.05 to 0.57) across sites and seasons and may reflect unmeasured exposures to potential sources not related to water (e.g., occupational exposures, pharmaceuticals, consumer products, bleach-based cleaning agents, ambient air).

We saw comparable mean baseline blood ΣTHM levels in sites 1 and 3 (20–23 ng/L). Despite much lower THM concentrations in tap water, the mean blood ΣTHM concentration among site 2 participants was roughly 60% of the means for sites 1 and 3. This was primarily due to the disproportionately high baseline blood TCM concentrations for site 2, as shown in a previous study of baseline blood THM levels among women using heavily brominated waters (Miles et al. 2002). In addition to potential exposure to TCM from non-water sources, the route of exposure can also be a critical determinant of internal THM levels. Miles et al. (2002) reported that blood and water concentrations were more closely correlated after high THM dose events such as showering. Rapid hepatic metabolism or elimination occurs for ingested THMs, but THM exposures from inhalation and dermal absorption are distributed directly to the blood (Blount et al. 2011; Leavens et al. 2007). Some of the differences detected for baseline blood THM levels may also reflect genetic variability or induction of metabolizing enzymes by alcohol, medications, or drugs, as some studies have noted a 50-fold variability in cytochrome P450 (CYP) 2E1 enzyme activity in humans (Stephens et al. 1994). The predominant route of metabolism for THMs is oxidation via CYP2E1 (Guengerich et al. 1991; Lilly et al. 1997), whereas secondary metabolic pathways include reductive dehalogenation via CYP2B1/2 and CYP2E1 (and possibly CYP1A2) (Allis and Zhao 2002; Tomasi et al. 1985) and glutathione conjugation via glutathione *S*-transferase theta (GSTT11) (DeMarini et al. 1997; Pegram et al. 1997). Genetic polymorphisms can vary widely by race and ethnicity; however, the preponderance of Caucasians in our sample population (69%) limited our ability to examine this as a potential contributing factor. Lack of data on medication use, occupational exposures, and use of consumer products precluded examination of the influence of these factors on blood THM levels.

In contrast to the 59% of brominated THMs detected in tap water across all three sites, we found lower proportions in blood (37%). Lower relative proportions of baseline brominated blood THMs have been observed in previous studies (Backer et al. 2000, 2008; Miles et al. 2002). These findings are consistent with known toxicokinetic differences between the individual THMs, as brominated THMs are more readily metabolized and are more lipophilic than TCM (Lilly et al. 1997). Partitioning of THMs from fat to blood and their subsequent metabolism were likely the predominant factors affecting baseline blood concentrations. Thus, slower release of brominated THMs from fat and more rapid primary metabolism would lead to lower baseline blood levels of brominated THMs compared with TCM. The molar rate of metabolism of the brominated THMs is greater than that of TCM (Lilly et al. 1997), and therefore, the brominated THM mass would decrease at a more pronounced rate compared with TCM. This shift toward less brominated species in blood may also be related to the exposure levels that women are experiencing from ambient air because more volatile species, such as TCM, are available for inhalation than are the more reactive brominated species. Dose levels from ambient air and drinking water exposures can also affect the metabolic pathway that is engaged. For example, brominated THMs are much more likely than TCM to proceed through secondary metabolic pathways, with GST-mediated conjugation of TCM to glutathione occurring only at extremely high TCM concentrations or doses (DeMarini et al. 1997; Pegram et al. 1997). As such, this may also help explain the higher relative TCM blood levels among site 2 participants with low tap water TCM concentrations. Given increasing epidemiological and toxicological evidence of health effects from DBPs being modulated by genetic polymorphisms (Cantor et al. 2010; DeAngelo et al. 1999; DeMarini et al. 1997; Kogevinas et al. 2010; Pegram et al. 1997), future studies should examine the variability in THM metabolism and clearance among susceptible populations.

The individual-level information collected on blood THM and residential tap water concentrations, water-use activities, and socio-demographic data among post-partum women was a strength of the study, but the small sample size limited our ability to fully elucidate independent contributors of blood THM concentrations. We had minimal repeated measures among participants to examine intraindividual differences, and data collected from only two seasons did not allow a more detailed assessment of temporal changes in baseline blood THM levels. We also had limited ability to examine the impact of episodic high THM dose events because few participants reported bathing (9%) and swimming (2%) in the 24-hr water-use diary. The women providing the convenience samples in our study were motivated and highly educated and represented a low-risk population. Although this non-random sample may not be representative of the general population, there is little reason to suspect that the relationships found here would not be applicable to other groups.

Despite these limitations, the detailed self-reported data allowed us to develop aggregate exposure metrics that addressed the multi-route exposure of volatile DBPs such as the THMs. We detected increasing blood THM concentrations across exposure metrics based on the noningestion metric alone or a total exposure metric based on ingestion and noningestion data. For example, the noningestion metric showed that women who spent more time engaging in water-use activities (i.e., showering and bathing oneself, bathing children, washing dishes) had higher blood ΣTHM levels. The liter-equivalents used here and in previous epidemiological studies (Dodds et al. 2004; King et al. 2004) were based on experimental studies of TCM (Kerger et al. 2000; Weisel and Jo 1996), but more recent findings by Haddad et al. (2006) and Valcke and Krishnan (2010) provide additional support for the equivalencies used to examine the relationship between showering and ingestion. The results of the sensitivity analyses indicate that the mean blood THM levels for the total exposure metric tertiles were not very sensitive to changes in equivalencies for activities largely driven by dermal absorption or inhalation. Overall, the total exposure metric, based on liter-equivalents, was not a strong predictor of baseline blood THM levels after adjusting for tap water ΣTHM concentrations and other sociodemographic variables.

In summary, we detected temporal and spatial variability in baseline blood THM concentrations in our study population. Water THM levels tended to vary more than blood THM levels in our population, and blood–water correlations for ΣTHM and individual THMs differed across sites and seasons. The blood–water correlations for the brominated THMs were much stronger than for TCM and more consistent across study locations. As noted previously, the TCM results could be more affected by metabolic differences, other non-water sources, or other factors that may vary across site or seasons. Our study results may prove useful in future epidemiological studies that examine THM exposure surrogates or that quantify the degree of bias from exposure measurement error, because the brominated THMs are more potent toxicants and carcinogens than TCM in experimental studies (Plewa et al. 2008) and thus are of greater concern for potential adverse health effects in humans. The results from our regression analysis also provide some support that tap water concentrations are predictive of baseline blood biomarker levels. THM water concentrations were also found to be an important predictor of post-shower blood THM levels in a previous study (Backer et al. 2008). Our multi-variate regression analyses indicated that the liter-equivalency–based metrics were not predictive of baseline blood THM concentrations; however, they still may hold some value at predicting peak blood THM levels in -epidemio-logical studies.

## Supplemental Material

(295 KB) PDF

## References

[r1] Allis JW, Zhao G (2002). Quantitative evaluation of bromodichloro-methane metabolism by recombinant rat and human cytochrome P450s.. Chem Biol Interact.

[r2] Ashley DL, Blount B, Singer PC, Depaz E, Wilkes C, Gordon S (2005). Changes in blood trihalomethane concentrations resulting from differences in water quality and water-use activities.. Arch Environ Occup Health.

[r3] Backer LC, Ashley DL, Bonin MA, Cardinali FL, Kieszak SM, Wooten JV (2000). Household exposures to drinking water disinfection by-products: whole blood trihalomethane levels.. J Expo Anal Environ Epidemiol.

[r4] Backer LC, Lan Q, Blount BC, Nuckols JR, Branch R, Lyu CW (2008). Exogenous and endogenous determinants of blood trihalomethane levels after showering.. Environ Health Perspect.

[r5] Blount BC, Aylward LL, LaKind JS, Backer LS, Hays SM (2011). Human exposure assessment for DBPs: factors influencing blood trihalomethane levels.. In: Encyclopedia of Environmental Health, Vol 3 (Nriagu JO, ed)..

[r6] Bonin MA, Silva LK, Smith MM, Ashley DL, Blount BC (2005). Measurement of trihalomethanes and methyl *tert*-butyl ether in whole blood using gas chromatography with high-resolution mass spectrometry.. J Anal Toxicol.

[r7] Bove FJ, Fulcomer MC, Klotz JB, Esmart J, Dufficy EM, Savrin JE (1995). Public drinking water contamination and birth outcomes.. Am J Epidemiol.

[r8] Cammann K, Hubner K. (1995). Trihalomethane concentrations in swimmers’ and bath attendants’ blood and urine after swimming or working in indoor swimming pools.. Arch Environ Health.

[r9] Cantor KP, Hoover R, Hartge P, Mason TJ, Silverman DT, Altman R (1987). Bladder cancer, drinking water source, and tap water consumption: a case-control study.. J Natl Cancer Inst.

[r10] Cantor KP, Villanueva CM, Silverman DT, Figueroa JD, Real FX, Garcia-Closas M (2010). Polymorphism GSTT1, GSTZ1, and CYP2E1, disinfection by-products, and risk of bladder cancer in Spain.. Environ Health Perspect.

[r11] Cardinali FL, Ashley DL, Morrow JC, Moll DM, Blount BC (2004). Measurement of trihalomethanes and methyl *tertiary*-butyl ether in tap water using solid-phase microextraction GC-MS.. J Chromatogr Sci.

[r12] Cardinali FL, McCraw JM, Ashley DL, Bonin M, Wooten J (1995). Treatment of vacutainers for use in the analysis of volatile organic compounds in human blood at the low parts-per-trillion level.. J Chromatogr Sci.

[r13] DeAngelo AB, George MH, House DE (1999). Hepato-carcino--geni-city in the male B6C3F1 mouse following a lifetime exposure to dichloroacetic acid in the drinking water: dose-response determination and modes of action.. J Toxicol Environ Health A.

[r14] DeMarini DM, Shelton ML, Warren SH, Ross TM, Shim JY, Richard AM (1997). Glutathione *S*-transferase-mediated induction of GC→AT transitions by halomethanes in *Salmonella*.. Environ Mol Mutagen.

[r15] Dodds L, King W, Allen AC, Armson BA, Fell DB, Nimrod C (2004). Trihalomethanes in public water supplies and risk of stillbirth.. Epidemiology.

[r16] Dodds L, King W, Woolcott C, Pole J. (1999). Trihalomethanes in public water supplies and adverse birth outcomes.. Epidemiology.

[r17] Forssén UM, Herring AH, Savitz DA, Nieuwenhuijsen MJ, Murphy PA, Singer PC (2007). Predictors of use and consumption of public drinking water among pregnant women.. J Expo Sci Environ Epidemiol.

[r18] Gordon SM, Brinkman MC, Ashley DL, Blount BC, Lyu C, Masters J (2006). Changes in breath trihalomethane levels resulting from household water-use activities.. Environ Health Perspect.

[r19] Grellier J, Bennett J, Patelarou E, Smith RB, Toledano MB, Rushton L (2010). Exposure to disinfection by-products, fetal growth, and prematurity: a systematic review and meta-analysis.. Epidemiology.

[r20] Guengerich FP, Kim DH, Iwasaki M (1991). Role of human cytochrome P450 IIE1 in the oxidation of many low molecular weight cancer suspects.. Chem Res Toxicol.

[r21] Haddad S, Tardif GC, Tardif R (2006). Development of physiologically based toxicokinetic models for improving the human indoor exposure assessment to water contaminants: trichloroethylene and trihalomethanes.. J Toxicol Environ Health A.

[r22] Hoffman CS, Mendola P, Savitz DA (2008). Drinking water disinfection by-products exposure and fetal growth.. Epidemiology.

[r23] Hornung RW, Reed LD (1990). Estimation of average concentration in the presence of nondetectable values.. Appl Occup Envir Hyg.

[r24] Hwang B-F Jaakkola JJK Guo H-R 2008 Water disinfection by-products and the risk of specific birth defects: a population-based cross-sectional study in Taiwan. Environ Health 7 23; doi: [Online 2 June 2008] 10.1186/1476-069X-7-23 18518952 PMC2467412

[r25] Kerger BD, Schmidt CE, Paustenbach DJ (2000). Assessment of airborne exposure to trihalomethanes from tap water in residential showers and baths.. Risk Anal.

[r26] King WD, Dodds L, Allen AC (2000). Relation between stillbirth and specific chlorination by-products in public water supplies.. Environ Health Perspect.

[r27] King WD, Dodds L, Armson A, Allen AC, Fell DB, Nimrod C (2004). Exposure assessment in epidemiologic studies of adverse pregnancy outcomes and disinfection byproducts.. J Exp Anal Environ Epidemiol.

[r28] Kogevinas M, Villanueva CM, Font-Ribera L, Liviac D, Bustamante M, Espinoza F (2010). Genotoxic effects in swimmers exposed to disinfection by-products in indoor swimming pools.. Environ Health Perspect.

[r29] Krasner SW, Weinberg HS, Richardson SD, Pastor SJ, Chinn R, Sclimenti MJ (2006). Occurrence of a new genera-tion of disinfection byproducts.. Environ Sci Technol.

[r30] Krasner SW, Wright JM (2005). The effect of boiling water on disinfection by-product exposure.. Water Res.

[r31] LaKind JS, Naiman DQ, Hays SM, Aylward LL, Blount BC (2010). Public health interpretation of trihalomethane blood levels in the United States; NHANES 1999–2004.. J Expo Sci Environ Epidemiol.

[r32] Leavens TL, Blount BC, DeMarini DM, Madden MC, Valentine JL, Case MW (2007). Disposition of bromodichloromethane in humans following oral and dermal exposure.. Toxicol Sci.

[r33] Lilly PD, Ross TM, Pegram RA (1997). Trihalomethane comparative toxicity: acute renal and hepatic toxicity of chloroform and bromodichloromethane following aqueous gavage.. Fundam Appl Toxicol.

[r34] Lynberg M, Nuckols JR, Langlois P, Ashley D, Singer P, Mendola P (2001). Assessing exposure to disinfection by-products in women of reproductive age living in Corpus Christi, Texas, and Cobb County, Georgia: descriptive results and methods.. Environ Health Perspect.

[r35] McGeehin MA, Reif JS, Becher JC, Mangione EJ (1993). Case-control study of bladder cancer and water disinfection methods in Colorado.. Am J Epidemiol.

[r36] Miles AM, Singer PC, Ashley DL, Lynberg MC, Mendola P, Langlois PH (2002). Comparison of trihalomethanes in tap water and blood.. Environ Sci Technol.

[r37] Nieminski EC, Chaudhauri S, Lemareaux T (1993). The occurrence of DBPs in Utah drinking water.. J Am Water Works Assoc.

[r38] Nuckols JR, Ashley DL, Lyu C, Gordon SM, Hinckley AF, Singer P (2005). Influence of tap water quality and household water-use activities on indoor air and internal dose levels of trihalo-methanes.. Environ Health Perspect.

[r39] Obolensky A, Singer PC (2005). Halogen substitution patterns among disinfection byproducts in the information collection rule database.. Environ Sci Technol.

[r40] Parvez S, Rivera-Núñez Z, Meyer A, Wright JM (2011). Temporal variability in trihalomethane and haloacetic acid concentrations in Massachusetts public drinking water systems.. Environ Res.

[r41] Pegram RA, Andersen ME, Warren SH, Ross TM, Claxton LD (1997). Glutathione *S*-transferase-mediated mutagenicity of trihalomethanes in *Salmonella typhimurium*: contrasting results with bromodichloromethane and chloroform.. Toxicol Appl Pharmacol.

[r42] Plewa MJ, Wagner ED, Muellner MG, Hsu KM, Richardson SD (2008). Comparative mammalian cell toxicity of N-DBPs and C-DBPs.. In: Occurrence, Formation, Health Effects (Karanfil T, Krasner SW, Westerhoff P, Xie Y, eds). ACS Symposium Series no. 995..

[r43] Porter CK, Putnam SD, Hunting KL, Riddle MR (2005). The effect of trihalomethane and haloacetic acid exposure on fetal growth in a Maryland county.. Am J Epidemiol.

[r44] Promislow JH, Makarushka CM, Gorman JR, Howards PP, Savitz DA, Hartmann KE (2004). Recruitment for a community--based study of early pregnancy; the Right from the Start study.. Paediatr Perinat Epidemiol.

[r45] Rahman MB, Driscoll T, Cowie C, Armstrong BK (2010). Disinfection by-products in drinking water and colorectal cancer: a meta-analysis.. Int J Epidemiol.

[r46] Savitz DA, Singer PC, Hartmann KE, Herring AH, Weinberg HS, Makarushka C (2005). Drinking Water Disinfection By-products and Pregnancy Outcome. Final Report for AwwaRF Project no 2579.

[r47] Savitz DA, Singer PC, Herring AH, Hartmann KE, Weinberg HS, Makarushka C (2006). Exposure to drinking water disinfection by-products and pregnancy loss.. Am J Epidemiol.

[r48] Singer PC (1994). Control of disinfection by-products in drinking water.. J Environ Eng.

[r49] Stephens EA, Taylor JA, Kaplan N, Yang CH, Hsieh LL, Lucier GW (1994). Ethnic variation in the CYP2E1 gene: polymorphism analysis of 695 African-Americans, European-Americans and Taiwanese.. Pharmacogenetics.

[r50] Toledano MB, Nieuwenhuijsen MJ, Best N, Whitaker H, Hambly P, de Hoogh C (2005). Relation of trihalomethane concentrations in public water supplies to stillbirth and birth weight in three water regions in England.. Environ Health Perspect.

[r51] Tomasi A, Albano E, Biasi F, Slater TF, Vannini V, Dianzani MU (1985). Activation of chloroform and related trihalomethanes to free radical intermediates in isolated hepatocytes and in the rat in vivo as detected by the ESR-spin trapping technique.. Chem Biol Interact.

[r52] Valcke M, Krishnan K. (2010). An assessment of the inter-individual variability of internal dosimetry during multi-route exposure to drinking water contaminants.. Int J Environ Res Public Health.

[r53] Villanueva CM, Cantor KP, Grimalt JO, Malats N, Silverman D, Tardon A (2007). Bladder cancer and exposure to water disinfection by-products through ingestion, bathing showering, and swimming in pools.. Environ Res.

[r54] Waller K, Swan SH, Windhan GC, Fenster L (2001). Influence of exposure assessment methods on risk estimates in an epidemiologic study of total trihalomethane exposure and spontaneous abortion.. J Expo Anal Environ Epidemiol.

[r55] Weinberg HS, Pereira VR, Singer PC, Savitz DA (2006). Considerations for improving the accuracy of exposure to disinfection by-products by ingestion in epidemiologic studies.. Sci Total Environ.

[r56] Weisel CP, Jo WK (1996). Ingestion, inhalation and dermal exposures to chloroform and trichloroethane from tap water.. Environ Health Perspect.

[r57] Williams DT, LeBel GL, Benoit FM (1997). Disinfection by-products in Canadian drinking water.. Chemosphere.

[r58] Wright JM, Schwartz J, Dockery DW (2004). The effect of disinfection byproducts and mutagenic activity on birth weight and gestation duration.. Environ Health Perspect.

[r59] Yang C-Y, Xiao Z-P, Ho S-C, Wu T-N, Tsai S-S (2007). Association between trihalomethane concentrations in drinking water and adverse pregnancy outcome in Taiwan.. Environ Res.

