# Supplementary material for: Comparison of Trihalomethanes in Tap Water and Blood: A Case Study in the United States
Source: Environ Health Perspect. 2012 Jan 26;120(5):661–7. doi: 10.1289/ehp.1104347 (PMC3346785; doi:10.1289/ehp.1104347)
Supplement: (295 KB) PDF [file ehp.1104347.s001.pdf]

**Supplemental Material**

# Comparison of Trihalomethanes in Tap Water and Blood: A Case Study in the United States

Zorimar Rivera-Núñez[1], J. Michael Wright[2]*, Benjamin C. Blount[3], Lalith K. Silva[3], Elizabeth Jones[4], Ronna L. Chan[5], Rex A. Pegram[6], Philip C. Singer[7], David A. Savitz[8]

[1]National Research Council, Washington, DC

[2]U.S. Environmental Protection Agency, National Center for Environmental Assessment, Cincinnati, OH

[3]Centers for Disease Control and Prevention, Atlanta, GA

[4]Association of Schools of Public Health, Washington DC

[5]University of North Carolina at Chapel Hill, Gillings School of Global Public Health, Department of Epidemiology, Chapel Hill, NC

[6]U.S. Environmental Protection Agency, National Health and Environmental Effects Research Laboratory, Research Triangle Park, NC

[7]University of North Carolina at Chapel Hill, Gillings School of Global Public Health, Department of Environmental Sciences and Engineering, Chapel Hill, NC

[8]Brown University, Departments of Community Health and Obstetrics and Gynecology, Providence, RI

*Corresponding Author: J. Michael Wright, U.S. Environmental Protection Agency, National Center for Environmental Assessment, 26 West Martin Luther King Drive, MS A-110, Cincinnati, OH 45268, 513-569-7922, wright.michael@epa.gov.

## Supplemental Material Table of Contents


Supplemental Material, Table 1. Characteristics based on first 24-hour water use diary data of a study population (n = 150) recruited from three metropolitan areas in the U.S. during 2004 and 2005. .......... S3

Supplemental Material, Table 2. Spearman correlation coefficients for blood and tap water THM concentrations by site and season. .......... S9

Supplemental Material, Table 3. Blood ΣTHM linear regression coefficients (per ng/L change) for different covariates among the study population ($n$ = 142). .......... S10

Supplemental Material, Figure 1. Study population based on blood, water, and questionnaire data inclusion criteria. .......... S13

Supplemental Material, Figure 2. Distribution of blood ΣTHM levels. .......... S14

Supplemental Material, Figure 3. Distribution of water ΣTHM levels. .......... S14

**Supplemental Material, Table 1.** Characteristics based on first 24-hour water use diary data of a study population (n = 150) recruited from three metropolitan areas in the U.S. during 2004 and 2005.

| Population Characteristics | *n* | % | Water Intake (L) | Shower/Bathing Self (minutes) | Bathing Children (minutes) | ΣTHM Blood (ng/L) | ΣTHM Water (µg/L)[a] |
|---|---|---|---|---|---|---|---|
| Total population | 150 | 100 | 0.90 | 16.4 | 8.5 | 18.7 | 17.2 |
| Maternal race/ethnicity | | | | | | | |
| Nonhispanic white | 104 | 69.3 | 0.98 | 13.4 | 8.3 | 18.7 | 17.0 |
| Nonhispanic black | 19 | 12.7 | 0.86 | 28.1 | 7.1 | 16.3 | 15.7 |
| Hispanic | 12 | 8.0 | 0.46 | 22.3 | 7.1 | 23.7 | 16.3 |
| Other | 7 | 4.7 | 0.63 | 15.2 | 6.7 | 18.5 | 17.4 |
| Missing | 8 | 5.3 | 0.74 | 18.9 | 18.8 | 19.0 | 29.0 |
| Maternal age (years) | | | | | | | |
| < 25 | 24 | 16.0 | 0.67 | 28.3 | 5.4 | 17.5 | 14.6 |

**Supplemental Material, Table 1 (cont.)**

| Population Characteristics | $n$ | % | Water Intake (L) | Shower/Bathing Self (minutes) | Bathing Children (minutes) | ΣTHM Blood (ng/L) | ΣTHM Water (µg/L)[a] |
|---|---|---|---|---|---|---|---|
| 25−29 | 52 | 34.7 | 0.69 | 15.0 | 6.6 | 20.1 | 18.2 |
| 30−34 | 49 | 32.7 | 1.20 | 12.7 | 6.9 | 19.7 | 17.3 |
| ≥ 35 | 17 | 11.3 | 1.20 | 12.5 | 18.5 | 14.2 | 14.4 |
| Missing | 8 | 5.3 | 0.74 | 18.9 | 18.8 | 19.0 | 29.0 |
| Highest maternal education level | | | | | | | |
| High school or less | 22 | 14.7 | 0.68 | 27.0 | 12.2 | 24.2 | 20.4 |
| Some college | 30 | 20.0 | 0.91 | 16.8 | 16.4 | 20.7 | 13.9 |
| College degree or higher | 90 | 60.0 | 0.96 | 13.5 | 13.5 | 17.0 | 17.1 |
| Missing | 8 | 5.3 | 0.74 | 18.9 | 18.8 | 19.0 | 29.0 |

**Supplemental Material, Table 1 (cont.)**

| Population Characteristics | $n$ | % | Water Intake (L) | Shower/Bathing Self (minutes) | Bathing Children (minutes) | ΣTHM Blood (ng/L) | ΣTHM Water (μg/L)[a] |
|---|---|---|---|---|---|---|---|
| Maternal smoking[b] | | | | | | | |
| Yes | 7 | 4.7 | 1.2 | 33.9 | 4.9 | 19.9 | 18.5 |
| No | 135 | 90.0 | 0.9 | 15.3 | 8.1 | 18.6 | 16.7 |
| Missing | 8 | 5.3 | 0.74 | 18.9 | 18.8 | 19.0 | 29.0 |
| Post-pregnancy BMI | | | | | | | |
| < 19.8 | 9 | 6.0 | 0.65 | 14.3 | 6.7 | 22.1 | 28.0 |
| 19.8−25.9 | 57 | 38.0 | 0.94 | 15.4 | 7.9 | 19.7 | 12.8 |
| 26.0−29.9 | 19 | 12.7 | 0.72 | 15.3 | 2.4 | 16.8 | 20.4 |
| > 29.9 | 34 | 22.7 | 1.10 | 17.4 | 10.7 | 19.6 | 20.0 |
| Missing | 31 | 20.7 | 0.77 | 18.4 | 11.4 | 16.5 | 19.8 |

**Supplemental Material, Table 1 (cont.)**

| Population Characteristics | *n* | % | Water Intake (L) | Shower/Bathing Self (minutes) | Bathing Children (minutes) | ΣTHM Blood (ng/L) | ΣTHM Water (µg/L)[a] |
|---|---|---|---|---|---|---|---|
| Marital status | | | | | | | |
| Married | 117 | 78.0 | 0.9 | 14.9 | 8.0 | 18.8 | 17.4 |
| Not married | 25 | 16.7 | 0.92 | 22.2 | 7.4 | 18.1 | 14.1 |
| Missing | 8 | 5.3 | 0.74 | 18.9 | 18.8 | 19.0 | 29.0 |
| Parity | | | | | | | |
| Nulliparous | 82 | 54.7 | 0.95 | 17 | 9.9 | 17.4 | 19.7 |
| Parous | 60 | 40.0 | 0.85 | 15.1 | 5.3 | 21.1 | 19.3 |
| Missing | 8 | 5.3 | 0.74 | 18.9 | 18.8 | 19.0 | 29.0 |
| Household income ($) | | | | | | | |
| < 30,000 | 31 | 20.7 | 0.94 | 21.5 | 7.8 | 21.1 | 16.5 |

**Supplemental Material, Table 1 (cont.)**

| Population Characteristics | *n* | % | Water Intake (L) | Shower/Bathing Self (minutes) | Bathing Children (minutes) | ΣTHM Blood (ng/L) | ΣTHM Water (μg/L)[a] |
|---|---|---|---|---|---|---|---|
| 30,001−60,000 | 41 | 27.3 | 0.86 | 16.2 | 8.4 | 22.3 | 19.0 |
| 60,001−80,000 | 34 | 22.7 | 0.73 | 16.1 | 6.9 | 15.7 | 17.1 |
| > 80,000 | 35 | 23.3 | 1.1 | 11.9 | 8.7 | 16.5 | 14.4 |
| Missing | 9 | 6.0 | 0.65 | 17.4 | 16.7 | 17.8 | 29.0 |
| Study site | | | | | | | |
| Site 1 | 70 | 46.7 | 0.86 | 14.9 | 9.6 | 23.1 | 24.8 |
| Site 2 | 49 | 32.7 | 0.99 | 17.1 | 7.7 | 12.6 | 4.8 |
| Site 3 | 31 | 20.7 | 0.81 | 18.3 | 7.3 | 23.5 | 24.8 |
| Water consumption[c] | | | | | | | |
| Tap water | 106 | 71 | 0.95 | 16.4 | 9.0 | 19.8 | 28.4 |

**Supplemental Material, Table 1 (cont.)**

| Population Characteristics | $n$ | % | Water Intake (L) | Shower/Bathing Self (minutes) | Bathing Children (minutes) | ΣTHM Blood (ng/L) | ΣTHM Water (µg/L)[a] |
|---|---|---|---|---|---|---|---|
| Bottled water | 8 | 5.3 | 0.64 | 15.2 | 7.0 | 21.1 | 27.1 |
| Tap and bottled water | 36 | 23.7 | 1.2 | 13.7 | 8.1 | 24.7 | 32.1 |

Abbreviations: THM, trihalomethane; TCM, chloroform; BDCM, bromodichloromethane; DBCM, dibromochloromethane; TBM, bromoform; ΣTHM, sum of TCM, BDCM, DBCM, and TBM. [a]Nine water samples were not examined due to unacceptable headspace volume and/or freezing of vials. [b]Any smoking during pregnancy. [c]Sample size reflects exclusive use of tap water, exclusive use of bottled water, or a combination of tap and bottled water. All variables reflect demographic characteristics during pregnancy except for post-pregnancy BMI. Blood and water THM concentrations below the limit of detection (LOD) were replaced with LOD/√2 for the analysis.

**Supplemental Material, Table 2.** Spearman correlation coefficients for blood and tap water THM concentrations by site and season.

| THMs | Overall (n = 150) | Site 1 Overall (n = 97) | Site 1 Winter (n = 47) | Site 1 Summer (n = 50) | Site 2[a] (n = 49) | Site 3[b] (n = 29) |
|---|---|---|---|---|---|---|
| ΣTHM | 0.36* | 0.12* | 0.03 | −0.25 | −0.04* | 0.51* |
| TCM | 0.37* | −0.02 | −0.14 | −0.24 | −0.05 | 0.57* |
| BDCM | 0.62* | 0.29* | 0.45* | −0.33* | 0.26* | 0.57* |
| DBCM | 0.53* | 0.44* | 0.51* | −0.11 | 0.45* | 0.33 |
| TBM | 0.54* | 0.09 | −0.23 | 0.07 | 0.14* | 0.38* |
| Brominated THMs | 0.53* | 0.35* | 0.44* | −0.20 | 0.20* | 0.44* |

Abbreviations: ΣTHM, sum of TBM, TCM, BDCM, and DBCM; TBM, bromoform; TCM, chloroform; BDCM, bromodichloromethane; DBCM, dibromochloromethane; brominated THMs, sum of BDCM, DBCM, and TBM. [a]All samples in Site 2 were collected during summer. [b]Only six samples in Site 3 were collected during summer. Concentrations below the limit of detection (LOD) were replaced with LOD/√2 for the analysis. *P-value for the Spearman rank correlation: $p < 0.05$.

**Supplemental Material, Table 3.** Blood ΣTHM linear regression coefficients (per ng/L change) for different covariates among the study population ($n$ = 142).

| Variables | Univariate β (SE) (ng/L) | Multivariate Model 1[a] β (SE) (ng/L) | Multivariate Model 2[b] β (SE) (ng/L) |
|---|---|---|---|
| ΣTHM water concentration (µg/L) | 0.206 (0.05)*** | 0.193 (0.05)*** | 0.190 (0.06)** |
| Age (years) | 0.025 (0.07) | 0.003 (0.08) | −0.045 (0.06) |
| Race/ethnicity[c] | 0.029 (0.07) | −0.018 (0.08) | 0.001 (0.09) |
| Education level[d] | −0.239 (0.08)** | −0.245 (0.11)** | −0.219 (0.12)* |
| Smoking (yes/no) | 0.010 (0.28) | 0.039 (0.34) | 0.062 (0.35) |
| Pre-pregnancy BMI[e] | 0.004 (0.06) | 0.004 (0.07) | 0.078 (0.07) |
| Marital status[f] | −0.403 (0.17)* | −0.476 (0.23)** | −0.418 (0.24)* |
| Income[g] | −0.094 (0.06) | −0.068 (0.08) | −0.080 (0.08) |
| Season[h] | −0.014 (0.13) | −0.015 (0.16) | −0.015 (0.17) |
| Site 1 vs. Site 2 | 0.021 (0.09) | 0. 023 (0.07) | 0.023 (0.08) |

**Supplemental Material, Table 3 (cont.)**

| Variables | Univariate | Multivariate Model 1[a] | Multivariate Model 2[b] |
|---|---|---|---|
| | β (SE) | β (SE) | β (SE) |
| Site 1 vs. Site 3 | 0.034 (0.08) | 0.051 (0.10) | 0.048 (0.10) |
| Noningestion metric (minute/day) | 0.002 (0.001) | 0.002 (0.004) | – |
| Ingestion metric (L/day) | 0.011 (0.01) | < 0.001(0.002) | – |
| Shower/Bathing (minute/day) | −0.001 (0.01) | – | −0.003 (0.006) |
| Children bath (minute/day) | 0.003 (0.004) | – | 0.007 (0.007) |
| Post-shower/bath bathroom time (minute/day) | 0.005 (0.0001) | – | 0.001 (0.005) |
| Dishwashing by hand (minute/day) | < 0.001 (0.001) | – | 0.000 (0.009) |
| Swimming (minute/day) | 0.007 (0.01) | – | 0.001 (0.01) |
| Tap water intake (L/day) | −0.034 (0.08) | – | 0.403 (0.09) |

Abbreviations: SE, standard error. [a]Ingestion vs. noningestion metrics. [b]Individual activities. [c]Nonhispanic white vs. all other races combined. [d]Some college or higher vs. high school or less. [e]Body Mass Index before pregnancy (continuous). [f]Reference group is

“Married”. [g]US$ (continuous). [h]Winter vs. summer. Concentrations below the limit of detection (LOD) were replaced with LOD/√2 for the analysis. Two-tailed *p*-value for t-statistics: *$p < 0.10$; **$p < 0.05$; ***$p < 0.001$.

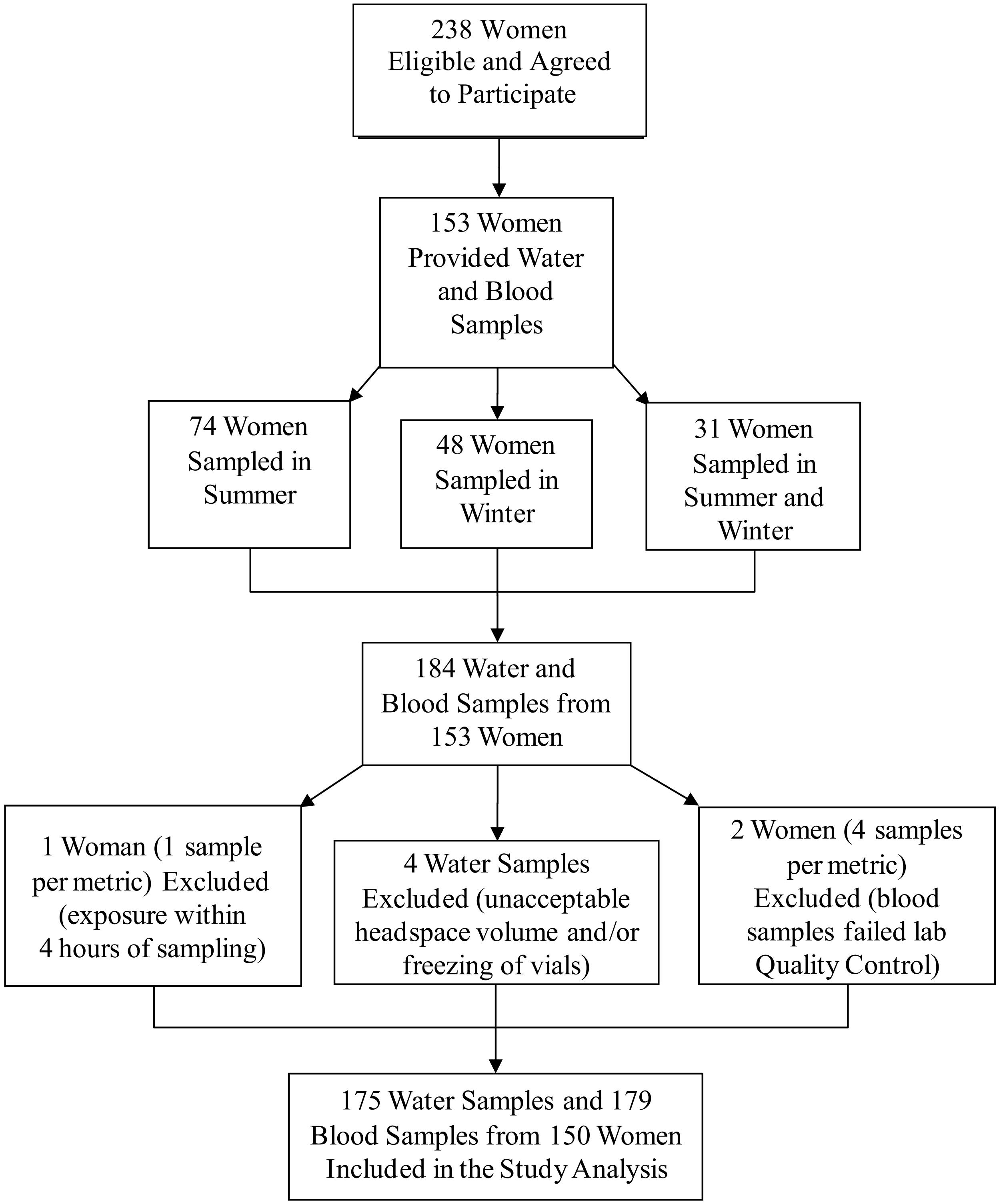


**Supplemental Material, Figure 1. Study population based on blood, water, and questionnaire data inclusion criteria.**

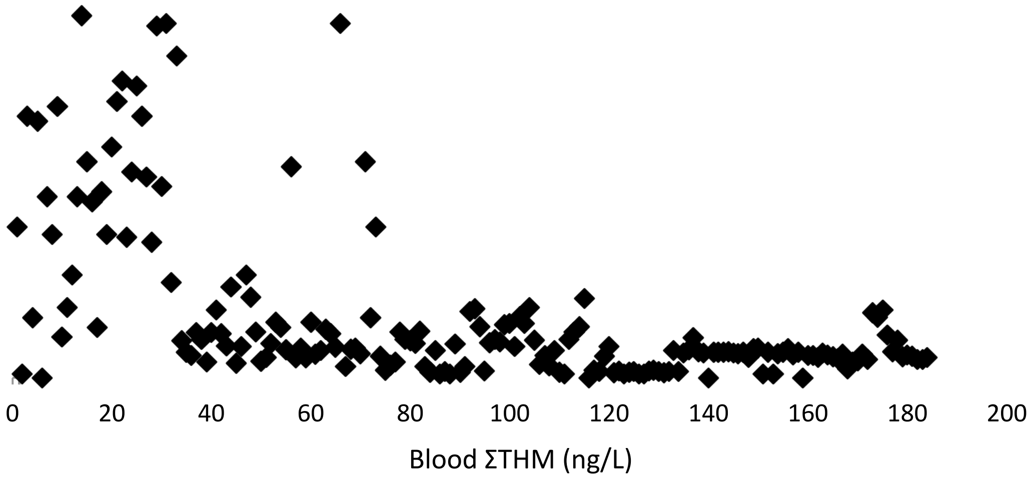


**Supplemental Material, Figure 2.** Distribution of blood ΣTHM concentrations.

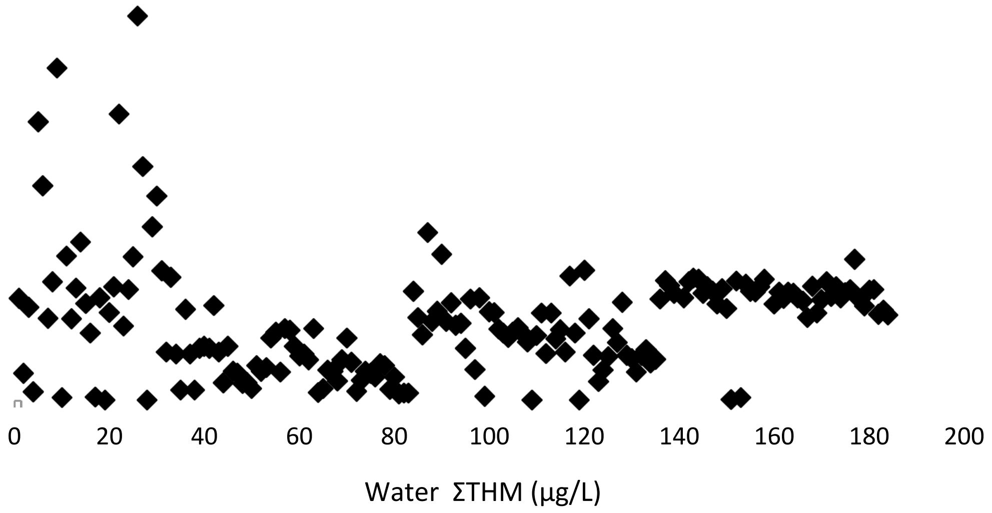


**Supplemental Material, Figure 3.** Distribution of water ΣTHM concentrations.
